# Longitudinal Optical Coherence Tomography Imaging Reveals Hyperreflective Foci Characteristics in Relapsing–Remitting Multiple Sclerosis Patients

**DOI:** 10.3390/jcm13175056

**Published:** 2024-08-26

**Authors:** Mathias Falck Schmidt, Gorm Pihl-Jensen, Michael Larsen, Jette Lautrup Frederiksen

**Affiliations:** 1 Department of Neurology, Clinic of Optic Neuritis, The Danish Multiple Sclerosis Center (DMSC), Rigshospitalet and University of Copenhagen, Valdemar Hansens Vej 13, 2600 Glostrup, Denmark; gorm.pihl-jensen@regionh.dk (G.P.-J.); jette.lautrup.battistini@regionh.dk (J.L.F.); 2 Department of Ophthalmology, Rigshospitalet and University of Copenhagen, 2600 Glostrup, Denmark; lars.michael.larsen@regionh.dk

**Keywords:** optical coherence tomography, relapsing-remitting multiple sclerosis, outer nuclear layer, hyperreflective foci, retinal infiltration, optic neuritis

## Abstract

**Background/Objectives:** Retinal hyperreflective foci, 25–50 µm in diameter, that can be imaged by noninvasive optical coherence tomography (OCT) may represent microglial activity related to inflammation. This study aimed to detect hyperreflective foci in the OCT-hyporeflective avascular outer nuclear layer of the retina in relapsing–remitting MS (RRMS) patients without ongoing eye or optic nerve disease. **Methods:** A cohort of 13 RRMS patients (8 eyes with and 18 eyes without prior optic neuritis) underwent retinal OCT at baseline, after 1 month, after 6 months, and then every 6 months for 3 years. The data were compared with single-examination data from 106 eyes in 53 age-matched healthy subjects. **Results:** The prevalence of hyperreflective foci at baseline was higher in RRMS patients than in healthy subjects (46.2% vs. 1.8%, *p* < 0.005). Patients with optic neuritis had much more foci than those without (*p* < 0.001). Hyperreflective foci recurred in 23.1% of RRMS patients, bilaterally in one with prior optic neuritis and unilaterally in two without. **Conclusions:** Patients with RRMS, notably those with prior optic neuritis, had elevated rates of retinal infiltration in the absence of retinal disease, suggesting that the phenomenon may represent elevated activity of an immune surveillance or housekeeping mechanism rather than retinal disease.

## 1. Introduction

Optical coherence tomography (OCT) is a non-invasive imaging method based on visible or infrared photonic radiation for non-invasive imaging of the clear and semi-opaque components of the eye. An occasional finding of uncertain significance is small punctate hyperreflective elements in the retina, called dots or foci (plural of focus), because they are too small for any internal variation in reflectivity to be discerned with current techniques [1]. Their role in retinal biology and pathology is largely unknown, but they aggregate locally in certain diseases, such as age-related macular degeneration [2] and central serous chorioretinopathy, and can disappear with the resolution of the disease [3]. Hyperreflective foci (HF) can be difficult to discern from blood vessels in cross-section and from the highly reflective retinal nerve fibers and plexuses, but they stand out with high contrast in the avascular hyporeflective outer nuclear layer (ONL). Single HF typically have a diameter of 30 µm or less, they do not cast a shadow, and they are not visible on fundus photographs, neither in color nor in infrared [4]. HF have been detected in various eye diseases and, to a lesser extent, in neurological diseases [5,6,7,8,9,10,11,12,13,14].

The histopathological correlation of HF is uncertain. Since they occur in the absence of exudative processes, they are unlikely to be hard exudate, i.e., plasma components that precipitate at a distance from their point of exudation where plasma components that can be resorbed by healthy vessels have been resorbed [15]. Instead, inferences from clinical observation and histopathology suggest that they may be macrophages or microglia that have become hyperreflective from accumulating ingested matter [16] or migrating retinal pigment epithelium cells [17,18]. Their occasional presence in patients with multiple sclerosis (MS) [12] has led to investigations of their presence in the inner retina of MS patients [13,14]. This exploration becomes particularly relevant given that numerous major neurodegenerative disorders manifest in the retina, reinforcing the concept of the retina as a ‘window’ into the brain [19]. 

MS is considered a chronic autoimmune disorder affecting the central nervous system (CNS), with the most common form being relapsing–remitting MS (RRMS), characterized by episodes of neurological decline followed by periods of recovery [20]. In RRMS, microglia, the CNS’s immune cells, release proinflammatory cytokines and neurotoxic substances, exacerbating inflammation, demyelination, and axonal damage, leading to neurological deficits [21,22].

Here, we present data on the presence and flux of hyperreflective elements in the ONL of the retina in MS patients without eye disease, where the scarcity of such foci allows for the manual counting of retinal HF over repeated visits. The observations were compared to cross-sectional data from healthy subjects. The working hypothesis of the study was that MS is accompanied by elevated proinflammatory activity in unmyelinated parts of the CNS.

## 2. Materials

The MS-related data are from a 3-year prospective multi-center non-interventional study of OCT in relapsing–remitting multiple sclerosis (RRMS) patients. Criteria for inclusion were having a diagnosis of RRMS as defined by the 2005 revision to the McDonald criteria [18], a duration of MS of more than 1 year from the time of diagnosis. Criteria for exclusion are listed in Supplementary Materials File S1. The research was carried out in accordance with the principles set forth in the Declaration of Helsinki and received approval from the medical ethics committee of the Greater Copenhagen Region. In total, 13 participants (median age 41 years (range 23–65)) were examined eight times between 29 May 2012 and 24 July 2017. Four of the 13 patients were diagnosed with ON at least 1 year before study entry. Baseline demographics and clinical characteristics are listed in Table 1.

## 3. OCT Scan Protocols

OCT was made using a Spectralis HRA plus OCT instrument (HEYEX recording software 1.9.10.0, version of 6.0.9.0, and viewing software 1.6.2.0, Heidelberg Engineering, Heidelberg, Germany). The examination of RRMS patients included peripapillary scans with a diameter of 12 degrees with automatic real-time averaging of 100 raw scans per line and a 20 × 20 degree macula block consisting of 25 vertical B-scans with an average of 49 scans per line and a line spacing of 240 µm. Scans were made at baseline, after 1 month, at 6 months and then every 6 months for 3 years, resulting in a total of 8 examinations, with follow-up scans automatically aligned with previous scans. Healthy subjects were examined once using peripapillary scans of 15-degree diameter with ART set at 30 and a fovea-centered 30 × 25 macular block of 120 horizontal B-scans with ART set at 30 and a line spacing of 60 µm.

Graders were blinded to information about clinical status other than group assignment, which followed from the difference in scan orientation. Analysis was made only of the macula scans, which are relatively free from shadowing from overlying vessels compared to the peripapillary scans.

## 4. Data Analysis

Scan analysis and HF counting were purposely limited to the ONL of the retina. All B-scans were reviewed by a single examiner (MFS), who manually identified all well-defined HF in the ONL of all eyes. The ONL was defined as the layer between the outer plexiform layer (OPL) and the photoreceptor layer as they present on an OCT scan, which has a profound hyporeflectivity compared to the adjacent layers. A hyperreflective focus was eligible if it was (i) located in the ONL, (ii) had the largest linear dimension ≤30 μm, (iii) did not cast a shadow, and (iv) had a reflectivity similar to that of the retinal nerve fiber layer (RNFL) in the same scan [23,24]. To avoid confusion with border region irregularities, counts included only foci with a distance from the adjacent layer exceeding the diameter of the focus. The quality of each OCT scan was evaluated using OSCAR-IB criteria [25]. The findings are presented in accordance with the Advised Protocol for OCT Study Terminology and Elements (APOSTEL) [26].

### 4.1. Hyperreflective Foci Distribution Density

**Figure 1:**
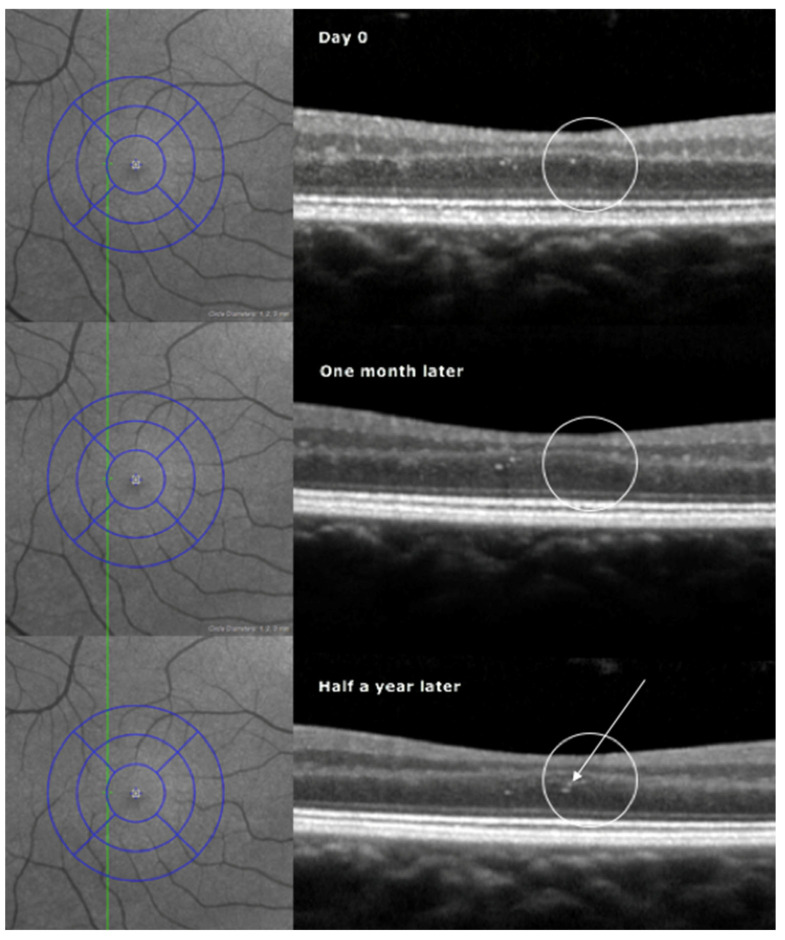
Infrared fundus image (**top left**) with an overlaid ETDRS grid in blue and a vertical OCT scan line in green. The baseline OCT (**top right**) shows a hyperreflective focus in the outer nuclear layer of the retina (encircled). One month later (**middle**), no hyperreflective element was seen. After half a year counted from baseline (**below**), a hyperreflective focus was found again at the same location, now together with three closely adjacent foci. Another cluster is seen to the left of the encircled area, followed by three, then two, and finally one hyperreflective element during the same period.

The *density* of HF in the ONL of the macula in MS patients was determined by marking and counting all foci in the ONL within a fovea-centered 6 × 6 mm^2^ area covered by the 25 vertical B-scans set at intervals of 240 µm. The Early Treatment Diabetic Retinopathy Study (ETDRS) grid, which was used as an overlay, consists of three concentric rings of diameters 1, 3, and 6 mm and two reticules that divide the macula into nine sections (Figure 1). The volume of the macula was calculated as the sum of the nine grid sections. The density of foci in healthy subjects was calculated by extracting a comparable area of the fundus from the block of horizontal scans. No compensation was made for variations in fundus magnification.

### 4.2. Focus-Tracking across Visits

A topographic evaluation was made with reference to the concentric rings of the ETDRS grid. To be accepted as a recurring observation of a hyperreflective focus at the same location, it had to be found on the same B-scan line at two or more consecutive visits within 15 µm of a previously observed focus (Figure 1).

## 5. Statistical Analyses

Guidance on the appropriate methodology, data preprocessing, and model specifications was provided by a statistician at the Faculty of Health and Medical Sciences. Statistical analysis was performed using SPSS Statistics, Version 24.0. (Armonk, NY, USA: IBM Corp.).

The number of foci between independent groups was compared using the Mann–Whitney U test according to distributions, while analyses involving categorical data (e.g., subjects with or without foci) were conducted using the Chi-Square and Fisher’s exact tests. The analyses mentioned above were conducted using data from both eyes. The results are presented as medians (including ranges). The level of statistical significance was set at 0.05.

## 6. Results

A total of 4900 B-scans were obtained across all patients and visits (both eyes were examined at every visit). A cross-sectional analysis was performed at baseline, comparing a dataset comprising 12,720 B-scans from 106 eyes of 53 healthy subjects.

**Figure 2:**
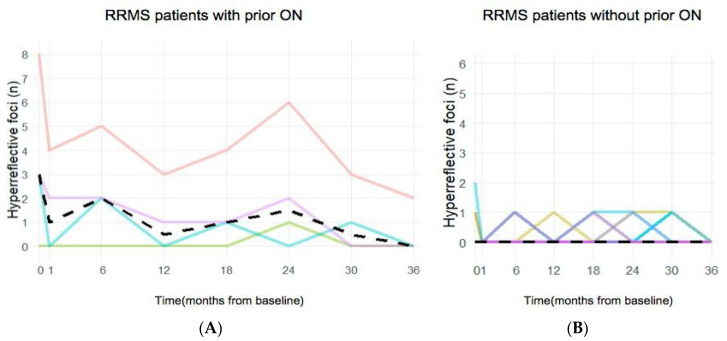
(**A**): The graph on the left illustrates the total number of hyperreflective foci (HF) observed in the outer nuclear layer (ONL) of the retina during each visit over the 3-year study period for relapsing–remitting multiple sclerosis patients with a prior episode of optic neuritis (RRMS + ON). (**B**): The graph on the right depicts the total number of HF in the ONL of the retina during each visit over the 3-year study period for RRMS patients without a history of ON (RRMS − ON). Each patient is depicted by a differently colored line, and the median number of hyperreflective foci is represented by a dashed black line. Consistently higher numbers of HF per visit were observed upon analysis in RRMS +ON patients over the 3-year study period (*p* < 0.05). *n* = number of HF.

HF in the ONL were found in 38.5% of RRMS patients, but only in 1.8% of healthy subjects (*p* < 0.005). Compared to RRMS patients without optic neuritis (RRMS −ON), patients with ON (RRMS +ON) exhibited a higher number of HF (*p* < 0.05 for six out of eight visits during the 3-year study period (Figure 2, statistics in Supplementary Materials File S2). The total foci count during the 3-year study period was higher in the RRMS +ON group compared to RRMS −ON, with respective medians of 9 (range 1–35) and 2 (range 0–5) (*p* < 0.001). In 3 out of 13 RRMS patients (23.1%), a location where a solitary HF was seen was empty at a subsequent visit and then presented a hyperreflective focus again within 15 µm of the initial location. HF were found bilaterally in one patient with a prior history of ON and unilaterally in two patients, one with and one without previous ON. In total, HF were observed bilaterally in one patient and unilaterally in eight patients (Supplementary Materials File S2). Cases of ON prior to study entry were all unilateral and uniformly in the right eye.

At baseline, the average number of HF per macular volume within the ONL (mm3) was 0.16 for the entire RRMS cohort and 0.005 in healthy subjects (*p* < 0.0001). The total number of new HF observed in RRMS patients during the 3-year period was 71, with 91.5% (65 out of 71) being situated within 6 mm of the center of the macula. There was no significant change in the fraction of RRMS patients who had HF at any given visit during the period of observation. At baseline, the number HF per unit B-scan length was notably higher in patients compared to healthy subjects (*p* > 0.001, Mann–Whitney U), with a median value of 0 (range 0–0.1).

## 7. Discussion

**Figure 3:**
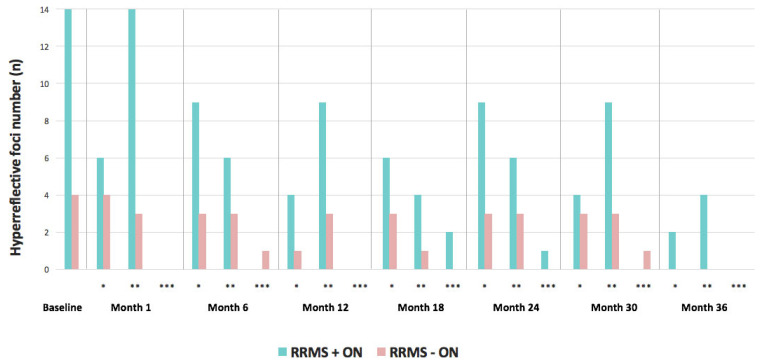
Hyperreflective foci number per visit per RRMS subgroup (never optic neuritis (red, *n* = 9), prior optic neuritis (green, *n* = 4)) divided into present at baseline and from then on into incident (*), gone (**), and recurrent (***).

Retinal infiltration with HF was rare in all study groups but nevertheless more common in RRMS patients than in healthy subjects, and to a higher degree among RRMS patients with prior ON compared to those who never had ON (Figure 2 and Figure 3). The presence and relative abundance of HF in each study subgroup showed no overall trend during the study, but fluctuations in the number of eyes with HF were noted within the RRMS group at two specific visits over the three-year observation period.

The findings of this study are consistent with a previous study of ours [11], which demonstrated that although HF in the ONL of the retina are rare in both RRMS patients and healthy subjects, compared to retinal diseases such as age-related macular degeneration and central serous chorioretinopathy [1], they are more frequently observed in RRMS patients than in healthy subjects.

Our observations suggest that HF in the retina are present after ON at a rate that exceeds background levels in healthy subjects.

Having a recurrent hyperreflective focus in the ONL that disappeared and reappeared at the same topographical location in the retina was associated with RRMS patients with prior ON. In these patients, there were no indications of genetic predispositions, lifestyle risk factor exposure, or disease other than MS and ON that could account for the presence of HF.

Upon activation, recruited macrophages have been shown to infiltrate the retina via the optic nerve and undergo morphological and functional characteristics that make them appear like resident microglia [27,28]. It is recognized that activated microglia can undergo a migration process in the retina, moving from the ganglion cell layer, inner plexiform layer, and OPL to the subretinal space above the retinal pigment epithelium [29,30]. We cannot exclude the possibility that our findings depict a retinal infiltration process that could be more prevalent among RRMS patients with a history of ON.

The origin of retinal HF is uncertain. It is of interest to determine if they are evidence of ongoing tissue attack with resulting tissue loss, if they merely represent a state of heightened immune alertness, or if they are evidence of a beneficial response that promotes healing in the CNS. Numerous retinal degenerative diseases and neurodegenerative disorders affecting vision are characterized by a lasting, chronic proinflammatory environment [31]. Microglia are commonly activated during inflammatory responses in neurodegenerative conditions such as MS [32]. The majority of acute inflammatory lesions in MS typically undergo transition into an inactive state, referred to as chronic inactive lesions, and diminish in size as a result of gliosis in the CNS. This persistent inflammation is believed to contribute to the formation of chronic active lesions, also known as “mixed active–inactive” or “smouldering” lesions [33,34,35]. Future studies should explore if this reflects a reactive state of gliosis in chronically active CNS lesions.

The methodological limitations of this study include its relatively small patient sample size and the wide spacing of scans compared to modern block scans tailored for en face viewing, which may capture a given hyperreflective focus on more than one B-scan. Another methodological constraint is the gradual transition between the ONL and the OPL that shifts with the angle of viewing of the OCT instrument, which may affect the contrast with which foci can be detected [36]. The advantages of the study technique include the high quality of the multiply averaged B-scans, the high number of visits, and the long, systematic follow-up. Other methodological strengths include the prospective design, the large number of high-resolution scans, and the meticulous grading of all scans by an experienced human grader, a procedure that has previously been shown to have high intra- and inter-grader repeatability in finding HF [18].

In this study, the OCT protocols for patients and healthy subjects featured slightly different recording parameters, particularly the length of each B-scan. With due correction for these differences the density of HF per unit B-scan length was consistently higher in patients than in control subjects throughout the study. Gender differences between patients and healthy subjects are also acknowledged. However, our study primarily examines the impact of RRMS and ON, and due to the small sample size, the study would lack the statistical power necessary to draw meaningful conclusions about gender-specific differences.

In conclusion, retinal infiltration with HF was more frequently observed in patients with RRMS than in healthy subjects, even in the absence of retinal disease. This prevalence was significantly higher among RRMS patients with a prior history of ON compared to those without.

To the best of our knowledge, this is the first study to longitudinally map HF in the avascular ONL of the retina in RRMS patients with and without ON. While it remains unclear whether HF represent a distinct pathological phenomenon or a variant of a more common occurrence within the CNS, their transient presence in the ONL may be attributed to elevated activity of an immune surveillance or housekeeping mechanism rather than an indication of retinal pathology. Future research should specifically investigate whether HF correlate with MS severity and progression, as this could offer valuable insights into the diagnostic and prognostic clinical value of HF in MS. Finally, more extensive studies are required before OCT, as a non-invasive method, can be applied on a larger scale with shorter intervals, to better define the dynamic characteristics of HF in MS patients.

## Figures and Tables

**Table 1:** Baseline demographics and clinical characteristics of RRMS, RRMS −ON, RRMS +ON patients and healthy subjects.

	RRMS Cohort (*n* = 13)	RRMS − ON (*n* = 9)	RRMS + ON (*n* = 4)	Healthy Subjects (*n* = 53)
Gender	92.2% female 7.8% male	88.9% female 11.1% male	100% female	56.6% female 43.4% male
Median age (range)	41 (23–65)	39 (33–62)	42 (29–65)	45 (20–67)
Median EDSS score (range)	2.0(0–4.5)	2.0(0–4.5)	2.7 (1–3.5)	NA
MS disease duration before OCT acquisition (range in years)	3 (1–5)	3 (1–5)	4 (2–6)	NA
Number of patients with and without				
hyperreflective foci in the outer nuclear layer				
*n* present (percentage)	6 (46.2%)	3 (33.3%)	3 (75%)	1 (1.8%)
*n* absent (percentage)	7 (53.8%)	6 (66.7%)	1 (25%)	52 (98.2%)
Median (range) of hyperreflective foci number	1 (0–8)	0 (0–2)	1 (0–6)	0 (0–2)
Ratio of hyperreflective foci (percentage)	71 out of 71	17 out of 71 (23.9%)	54 out of 71 (76.1%)	
Hyperreflective foci per macular volume within the entire retina (mm^3^)	0.16	0.12	0.21	0.005

Demographic and clinical characteristics for the entire cohort of relapsing–remitting multiple sclerosis (RRMS) patients, for the patient subgroups without any current or prior optic neuritis (RRMS − ON) or with a history of optic neuritis prior to study entry (RRMS + ON), and healthy subjects. The number of patients at baseline with and without hyperreflective foci (HF) present in the outer nuclear layer is shown for the two patient groups and control subjects. Corresponding medians for the total number of HF shown at baseline in separate patient subgroups (foci count per patient). Ratios show the number of HF within subgroups relative to the total number of foci ever observed in the RRMS cohort during the 3-year study. *n* = number of patients; NA: Not applicable.

## Data Availability

The original contributions presented in the study are included in the article and Supplementary Materials; further inquiries can be directed to the corresponding author.

## Supplementary Materials

The following supporting information can be downloaded at: https://www.mdpi.com/article/10.3390/jcm13175056/s1, Supplementary Materials File S1; Supplementary Materials File S2.
