# Supplementary material for: Longitudinal Optical Coherence Tomography Imaging Reveals Hyperreflective Foci Characteristics in Relapsing–Remitting Multiple Sclerosis Patients
Source: J Clin Med. 2024 Aug 26;13(17):5056. doi: 10.3390/jcm13175056 (PMC11396612; doi:10.3390/jcm13175056)
Supplement: Supplementary file 1 [file jcm-13-05056-s001.zip › jcm-3111536-Supplementary materials 1.pdf]

Supplementary materials_1

Exclusion criteria for participants in both the relapsing- remitting multiple sclerosis (RRMS) group (with and without optic neuritis) and healthy controls

- Retinal conditions associated with edema, cysts, subretinal fluid, etc.
- Stargardt disease
- pathologic myopia
- Age-related macular degeneration
- Uveitis and intraocular inflammatory disorders
- Glaucoma
- HIV or any other known immunodeficiency syndrome

Any ocular or systemic conditions, aside from RRMS and optic neuritis, that may impact the visual system encompassing:

- Systemic hypertension
- Diabetes melliitus
- Systemic lupus erythematosus
- Sjögren Syndrome
- Sarcoidosis
- Systemic infections
