# Supplementary material for: Longitudinal Optical Coherence Tomography Imaging Reveals Hyperreflective Foci Characteristics in Relapsing–Remitting Multiple Sclerosis Patients
Source: J Clin Med. 2024 Aug 26;13(17):5056. doi: 10.3390/jcm13175056 (PMC11396612; doi:10.3390/jcm13175056)
Supplement: Supplementary file 1 [file jcm-13-05056-s001.zip › jcm-3111536-Supplementary materials 2.pdf]

Supplementary materials 2_ Counting hyperreflective foci in the outer nuclear layer of the retina in patients with relapsing- remitting multiple sclerosis (RRMS)_

									Total number of HF	HF that disappears	HF that disappears – reappears
No.1	Baseline**	Month 1**	Month 6**	Month 12	Month 18**	Month 24**	Month 30	Month 36**			
Right eye	1	0	0	0	0	0	0	0	1 (1macula)	1	
Left eye	0	0	0	0	0	0	0	0			
No.2	Baseline**	Month 1**	Month 6**	Month 12	Month 18**	Month 24**	Month 30	Month 36**			
Right eye	6	4	3	3	1	3	2	2	35 (35 macula)	35	2
Left eye	2	0	2	0	3	3	1	0			
No.3	Baseline**	Month 1**	Month 6**	Month 12	Month 18**	Month 24**	Month 30	Month 36**			
Right eye	0	0	0	0	0	0	0	0			
Left eye	1	0	0	1	0	1	1	0	4 (4macula))	4	
No.4	Baseline**	Month 1**	Month 6**	Month 12	Month 18**	Month 24**	Month 30	Month 36**			
Right eye	0	0	0	0	0	1	0	0	1 (1macula)	1	
Left eye	0	0	0	0	0	0	0	0			
No.5	Baseline**	Month 1**	Month 6**	Month 12	Month 18**	Month 24**	Month 30	Month 36**			
Right eye	1	0	1	0	0	0	1	0	7 (7 macula)	7	1
Left eye	2	0	1	0	1	0	0	0			
No.6	Baseline**	Month 1**	Month 6**	Month 12	Month 18**	Month 24**	Month 30	Month 36**			
Right eye	0	0	0	0	0	0					
Left eye	0	0	0	0	0	0			0		
No.7	Baseline**	Month 1**	Month 6**	Month 12	Month 18**	Month 24**	Month 30	Month 36**			
Right eye	0	0	0	0	0	0	0				

Left eye	0	0	1	0	1	0	1		3 (3 macula)	3	
No.8	Baseline**	Month 1**	Month 6**	Month 12	Month 18**	Month 24**	Month 30	Month 36**			
Right eye	0	0	0	0	0	0	0	0			
Left eye	2	0	1	0	1	1	0	0	5 (5 macula)	3	1
No.9	Baseline**	Month 1**	Month 6**	Month 12	Month 18**	Month 24**	Month 30	Month 36**			
Right eye	0	0	0	0	0	0	0	0			
Left eye	0	0	0	0	0	0	1	0	1 (1macula)	1	
No.10	Baseline**	Month 1**	Month 6**	Month 12	Month 18**	Month 24**	Month 30	Month 36**			
Right eye	0	0	0	0	0	1	0	0			
Left eye	0	0	0	0	0	0	0	0	1 (1 macula)	1	
No.11	Baseline**	Month 1**	Month 6**	Month 12	Month 18**	Month 24**	Month 30	Month 36**			
Right eye	0	0	0	0	0	0					
Left eye	0	0	0	0	0	0			0	0	
No.12	Baseline**	Month 1**	Month 6**	Month 12	Month 18**	Month 24**	Month 30	Month 36**			
Right eye	0	0	1	0	1	0	0	0			
Left eye	0	0	0	0	0	0	0	0	2	2	
No.13	Baseline**	Month 1**	Month 6**	Month 12	Month 18**	Month 24**	Month 30	Month 36**			
Right eye	2	0	2	1	1	1	0	0	11 (11 macula)	2	
Left eye	1	2	0	0	0	1	0	0			

The numbers of high-intensity hyperreflective foci (HF) in the outer layer (ONL), defined as the region between the outer plexiform layer and photoreceptor layer were counted manually for right and left eye to each patient for each examination. HF that disappears in the ONL from their original location in the 3 year period are shown and HF that "appears-disappears" respectively on same original retinal location in the 3 year period.

*= higher number of HF observed in the RRMS +ON group compared to the RRMS -ON group (per individuals), $p < 0.05$

**= higher number of HF observed in the RRMS +ON group compared to the RRMS -ON group (per eyes), $p < 0.05$

Supplementary materials_2B_ Density of retinal hyperreflective foci in patients with relapsing- remitting multiple sclerosis (RRMS)

*= denotes the count of hyperreflective foci per macular volume (mm^3) during each visit.

** = represents the ratio of the cumulative count of hyperreflective foci ever observed during the entire three-year study period to the average macular volume (mm^3) across all visits.

									Hyperreflective foci number per mean macular volume (mm^3)
No.1	Baseline	Month 1	Month 6	Month 12	Month 18	Month 24	Month 30	Month 36	
Right eye	1 (0.11*)	0	0	0	0	0	0	0	0.11** (right eye)
Left eye	0	0	0	0	0	0	0	0	
No.2	Baseline	Month 1	Month 6	Month 12	Month 18	Month 24	Month 30	Month 36	
Right eye	6 (0.68*)	4(0.45*)	3(0.34*)	3(0.34*)	1(0.11*)	3(0.34*)	2(0.22*)	2(0.22*)	2.7** (right eye)
Left eye	2(0.22*)0.45	0	2(0.22*)	0	3(0.34*)	3(0.34*)	1(0.11*)	0	1.25**(left eye)
No.3	Baseline	Month 1	Month 6	Month 12	Month 18	Month 24	Month 30	Month 36	
Right eye	0	0	0	0	0	0	0	0	
Left eye	1(0.10*) 0.5	0	0	1(0.10*)	0	1(0.10*)	1(0.10*)	0	0.34** (left eye)
No.4	Baseline	Month 1	Month 6	Month 12	Month 18	Month 24	Month 30	Month 36	

Right eye	0	0	0	0	0	1 (0.11*)	0	0	0.11** (right eye)
Left eye	0	0	0	0	0	0	0	0	
No.5	Baseline	Month 1	Month 6	Month 12	Month 18	Month 24	Month 30	Month 36	
Right eye	1 (0.14*)	0	1 (0.14*)	0	0	0	1 (0.14*)	0	0.14** (right eye)
Left eye	2(0.28*) 0.21	0	1 (0.14*)	0	1 (0.14*)	0	0	0	0.18** (left eye)
No.6	Baseline	Month 1	Month 6	Month 12	Month 18	Month 24	Month 30	Month 36	
Right eye	0	0	0	0	0	0			
Left eye	0	0	0	0	0	0			
No.7	Baseline	Month 1	Month 6	Month 12	Month 18	Month 24	Month 30	Month 36	
Right eye	0	0	0	0	0	0	0		
Left eye	0	0	1(0.10*)	0	1(0.10*)	0	1(0.10*)		0.10** (left eye)
No.8	Baseline	Month 1	Month 6	Month 12	Month 18	Month 24	Month 30	Month 36	
Right eye	0	0	0	0	0	0	0	0	
Left eye	2(0.22*)	0	1(0.10*)	0	1(0.10*)	1(0.10*)	0	0	0.13** (left eye)
No.9	Baseline	Month 1	Month 6	Month 12	Month 18	Month 24	Month 30	Month 36	
Right eye	0	0	0	0	0	0	0	0	
Left eye	0	0	0	0	0	0	1 (0.11*)	0	0.11**(left eye)
No.10	Baseline	Month 1	Month 6	Month 12	Month 18	Month 24	Month 30	Month 36	
Right eye	0	0	0	0	0	0	0	0	
Left eye	0	0	0	0	0	0	1 (0.11*)	0	0.11**(left eye)
No.11	Baseline	Month 1	Month 6	Month 12	Month 18	Month 24	Month 30	Month 36	0
Right eye	0	0	0	0	0				

Left eye	0	0	0	0	0			
No.12	Baseline	Month 1	Month 6	Month 12	Month 18	Month 24	Month 30	Month 36	
Right eye	0	0	1(0.11*)	0	1(0.11*)	0	0	0	0.11**(right eye)
Left eye	0	0	0	0	0	0	0	0	
No.13	Baseline	Month 1	Month 6	Month 12	Month 18	Month 24	Month 30	Month 36	
Right eye	2(0.22*)	0	2(0.22*)	1(0.10*)	1(0.10*)	1(0.10*)	0	0	0.14** (right eye)
Left eye	1(0.10*)1	2(0.22*)	0	0	0	1(0.10*)	0	0	0.14** (left eye)

Supplementary materials_2C_Counting hyperreflective foci in the outer nuclear layer of the retina in healthy controls_

The numbers of high-intensity foci in the outer layers, defined as the region between the outer plexiform layer and photoreceptor layer were counted manually in both eyes of the 53 healthy controls (i.e. from blocks of B-scans set at a distance of 60µm). Only in one healthy control 2 foci were identified in the right eye (i.e. the total amount of hyperreflective foci per macular volume in the healthy subjects was 0.005 mm^3).
